# Of Mice and Men, and Chandeliers

**DOI:** 10.1371/journal.pbio.0060243

**Published:** 2008-09-23

**Authors:** Alan Woodruff, Rafael Yuste

## Abstract

How does the human neocortex reliably propagate information through neural circuits? One mechanism appears to involve relying on strong connections from pyramidal neurons to interneurons and a depolarizing action of cortical chandelier cells.

## Of Mice and Men

What makes us human? One of the most obvious answers to this age-old question lies in the structure and function of the central nervous system, particularly the neocortex, where unique human features may lie. In fact, humans have not only a proportionally much larger neocortex compared to that of other mammals, but also a huge frontal lobe, the font of higher cognition.

In seeking clues to the biological basis of being human, neuroanatomists have long compared the human brain to that of other species, leading them to develop two distinct theories. Santiago Ramón y Cajal, the father of neuroanatomy, argued that the cortex of “higher” mammals, like humans, has more classes of neurons than those of “lower” mammals, for which he used the mouse as an example [1]. Specifically, he proposed that the variety and sophistication of “short-axon” cells, i.e., GABAergic inhibitory interneurons, increases as one climbs up the evolutionary ladder [2].

The alternative position—that differences among species arise not from variations in cell types, but from the size and complexity of the circuits—was defended by Cajal's own disciple, Rafael Lorente de Nó, who, like many of the best students, did exactly the opposite of what he had been taught. Choosing the mouse as his experimental system for his thesis at the tender age of 20, Lorente described as many cell types in the mouse neocortex as Cajal had described in humans. Cajal politely published Lorente's paper in his journal without corrections [3], yet told his disciple that he was wrong. The argument would continue until Cajal's death: on his deathbed in 1934, Cajal wrote to Lorente, admonishing him: “the mouse is not a good choice for the study of cortical circuits because of its paucity of short-axon cells” [4].

## Of Chandelier Cells

**Figure 1:**
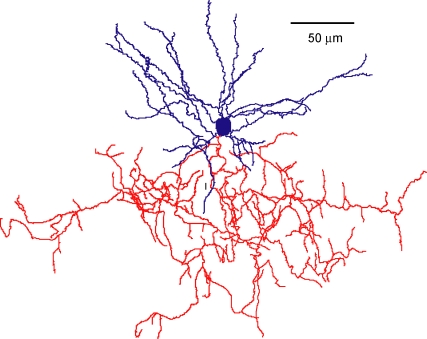
A Mouse Chandelier Cell Reconstruction of a biocytin-filled chandelier cell from a mouse neocortical brain slice. Soma and dendrites labeled in blue, axon arbor in red. Chandelier cells have characteristic terminal portions of their axon, which form short vertical rows of boutons resembling candlesticks. Each candlestick innervates a single axon initial segment of a pyramidal cell.

One of the most distinct types of short-axon cells, or GABAergic interneurons, present in mammalian cortical circuits is the “chandelier” cell. Their distinct axonal arbor, with parallel arrays of short vertical sets of presynaptic terminals (“cartridges”), resembles the candlesticks of an old-fashioned chandelier (Figure 1). Chandelier neurons are rare, forming only a small percentage of all GABAergic interneurons [5]—both Cajal and Lorente missed them—and were not described until 1974 by Szentagothai and Arbib [6]. A similar neuron with parallel arrays of terminals (type 4 cell) was reported by Jones at about the same time [7]. Based on the morphology of their terminals, Szentagothai thought that chandelier cells formed arrays of synapses with apical dendrites of pyramidal neurons [8], but this idea was proven wrong. Szentagothai's own disciple, Peter Somogyi, used electron microscopy to demonstrate that morphologically similar neurons, which he named axo-axonic cells (AACs), specifically contact the axon initial segment of pyramidal cells [9]. This key finding was confirmed by Fairen and Valverde [10] and DeFelipe et al. [11], who proposed that chandelier cells and AACs were the same cell type. Both terms have been used interchangeably in the literature ever since.

The striking morphologies of chandelier neurons have captured the imagination of cortical researchers and are often used as the best examples to illustrate the apparently purposeful design of cortical microcircuits. Each chandelier cartridge establishes a large number of synapses with each pyramidal neuron, strategically placed in the axon initial segment, where the action potential is generated. Thus, chandeliers appear ideally suited to shut off entire groups of pyramidal cells, making them the ultimate cortical switches.

Until recently, little was known about the function of chandelier cells, owing both to their rarity and the lack of unique neurochemical or physiological markers. Occasional recordings from chandelier cells in vitro [12,13] and in vivo [14,15] revealed their interneuronal firing properties. But two years ago, a landmark paper by Gabor Tamás and colleagues [16] turned the field upside down. Tamás, himself a disciple of Somogyi, argued that chandelier cells have an excitatory as well as an inhibitory function. Amazingly, a single action potential in a chandelier neuron could directly drive multiple postsynaptic pyramidal cells to spike, providing a high-fidelity mechanism for signal propagation in a local cortical microcircuit. Forcing pyramidal cells to spike could result in excitatory feedback on the chandelier cells, providing a physiological marker to distinguish at least some chandelier cells from other interneurons. A similar phenomenon of feedback excitation had likely been seen in hippocampal chandelier cells over a decade earlier [12], and was also recently described in the amygdala [17], suggesting that the excitatory role of chandelier cells may in fact be widespread.

To explain how chandelier cells could be excitatory, Tamás and colleagues argued that the GABA reversal potential (E_GABA_) is more depolarized at the axon than elsewhere in the neuron, due to the lack of the potassium chloride cotransporter KCC2, which extrudes chloride to the extracellular space. But simply lacking the cotransporter may be insufficient to maintain such a large E_GABA_ gradient between the axon and soma. Subsequently, a recent study utilizing GABA uncaging has reported a depolarizing shift in E_GABA_ from dendrite to soma to the axon, and further showed that high axonal expression of the chloride importer NKCC1, perhaps in addition to a lack of KCC2, could maintain a depolarized E_GABA_ [18].

## Of Human Chandeliers

In this issue of *PLoS Biology*, Molnár et al. extend their earlier findings on cortical chandelier cells, performing a technical tour de force. Recording from human surgical samples, the authors identify—for the first time in humans—pairs of connected neurons and study their synaptic and circuit properties [19]. Dual recordings from connected cells are the current “gold standard” of circuit neuroscience, because they allow physiological analysis of the effect of activating single axons [20,21], and thus the functional isolation of elementary, neuron-to-neuron, synaptic responses.

**Figure 2:**
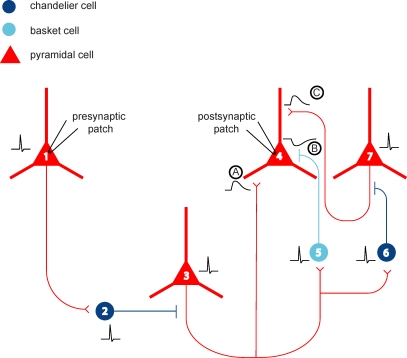
Hypothesized Propagation of Activity in Human Neocortex An action potential in a pyramidal neuron (cell 1) elicits a spike in a chandelier cell (2) via a strong connection, in turn evoking a third-order spike in a downstream pyramidal cell (3). This spike results in a trisynaptic EPSP being recorded in a postsynaptic pyramidal cell (cell 4, event A). At the same time, cell 3 drives both a basket cell (5) and chandelier cell (6) to threshold. The basket cell evokes a hyperpolarizing IPSP on the postsynaptically recorded pyramidal cell (cell 4, event B), four synapses removed from the original spike. The spiking chandelier cell (6) triggers yet another pyramidal neuron to fire (7), which produces an EPSP on the recorded neuron (cell 4, event C), five synapses away from the original spike. The result seen in the postsynaptic pyramidal neuron (cell 4) is a delayed EPSP-IPSP-EPSP sequence (events A, B, and C), traveling through three, four, and five synapses respectively. Molnár et al. propose that polysynaptic pathways similar to this one can be activated by a single action potential in a cortical pyramidal cell.

Molnár et al. show that a single action potential in a single layer 2/3 cortical pyramidal cell can trigger polysynaptic chains of activity, detected as excitatory postsynaptic potentials (EPSPs) and inhibitory postsynaptic potentials (IPSPs) in recorded neurons (Figure 2). This reveals an extremely efficacious means of activity propagation in the cortical network. Although earlier work had shown polysynaptic activations following a single chandelier spike [16,17], the current study demonstrates much longer responses. Moreover, one of the most interesting results from Molnár et al. relates to the temporal structure of the activity patterns elicited after stimulation of a single neuron. While most of them appear to propagate through the circuit with increasing disorganization, occasionally the authors were able to trigger an amazingly precise temporal pattern (see Figure 1B in Molnár et al.). This implies that the microcircuit is capable under some circumstances of generating patterns of activation with low jitter and high temporal precision, resembling precise spatiotemporal patterns of network activation reported in neocortical preparations in vivo and in vitro [22–24].

What is the mechanism of these activations? The authors identify two key factors. First, very strong (up to 8–20 mV) connections from pyramidal cells to basket and chandelier cells enable spike-to-spike transmission (Figure 2). In human brain slices, a relatively high proportion of basket (20%) and chandelier neurons (33%) could be driven to threshold by a single pyramidal neuron spike—in stark contrast to an estimated 1% likelihood of finding polysynaptic events in rat. The second factor involves chandelier cell recruitment of downstream pyramidal cells through their depolarizing effect. These downstream pyramidal cells may thus produce EPSPs three synapses removed from the original spike, while—by virtue of large-amplitude synapses—perhaps activating more chandeliers and basket cells (thereby producing a second round of IPSPs, this time four synapses downstream of the original spike!). Such polysynaptic chains of activation, alternating between pyramidal and axo-axonic cells, could theoretically continue unabated, except that synapses onto, and from, many interneurons exhibit synaptic depression (a decrease in synaptic strength [25,26]).

The finding of such strong synapses between pyramidal cells and interneurons in human samples raises many questions. While the authors rule out interspecies differences in interneuron input resistance in vitro, it remains plausible that in vivo input resistances are lower for humans than for other animals. In this case, the strikingly large synaptic amplitudes may reflect an adaptation to different electrotonic conditions. Also, given that the activation of pyramidal neurons by chandelier cells depends crucially on the resting membrane potential of the pyramidal cell and the chloride equilibrium potential at the axon initial segment, it is possible that the exact extracellular milieu in which the neurons are bathed could facilitate, or impede, this effect. In this respect, it is essential to repeat these rodent experiments in vivo. Also, the preparation of human surgical samples differs significantly from that of rodent brain slices, and it is possible that the reported interspecies differences result from the different methods used. Alternatively, the large-amplitude EPSPs that enable extended polysynaptic sequences could indeed be unique to humans. Finally, even a relatively distinct group of neurons such as chandelier/axo-axonic cells is composed of cells with different morphologies [11], so it is conceivable that different subtypes of chandeliers or AACs exist. Indeed, a recent report has described a new type of AAC [27], raising the possibility that equating chandelier cells and AACs may be too simplistic. Therefore, one needs to re-examine whether all AACs are chandelier cells and to establish whether the findings of Molnár et al. [19] and Tamás et al. [16] apply to all chandeliers or AACs, or only to a subtype of them.

## Significance and Future Directions

Regardless of these potential issues, the data stand in front of us, providing us a tantalizing glimpse into the functional microcircuitry of the human neocortex, as well as a new chapter in the fascinating exposition of the chandelier cell. Moreover, the results of Molnár et al. cut to the heart of circuit neuroscience, addressing the basic question of how activity propagates through a circuit. Specifically, their data contribute to three important problems:

*1. Role of single neurons in cortical networks:* Given that even a small volume of neocortex contains tens of thousands of neurons, and that excitatory synapses are generally weak, with depressing dynamics and low probability of success, the role of individual neurons in the cortex (or more generally, in the mammalian brain) is thought to be negligible. Rather, it is traditionally believed that only the joint activity of many neurons can rise above these biophysical limitations to have any functional impact. But recent data from the Brecht laboratory have challenged this basic assumption [28,29]. These in vivo experiments showed that the activation of individual neurons can alter the motor or sensory behavior of the animal, revealing the salience of single neuron in the brain. These remarkable experiments have lacked a mechanism that could explain how action potentials generated by a single neuron could ever propagate through these biophysical hurdles. Molnár et al. now provide a potential mechanism for the Brecht data: perhaps the stimulated neurons trigger the activity of chandelier cells, lighting up chains of activity. At the same time, the Brecht data were obtained in rats, so it would appear inconsistent with the low probability of generating polysynaptic chains in rat brain slices discussed by Molnár et al. Alternatively, there may be a difference in propagation efficacy in vitro versus in vivo. Nevertheless, the Molnár et al. data confirm that, at least in some circumstances and in some species, stimulation of individual cortical neurons in vivo can generate an activity pattern that propagates through the circuit.

*2. Generation of precise activity patterns:* A second significant contribution of Molnár et al. relates to the debate of whether the cortex can generate spatiotemporal patterns of activity with great accuracy. As mentioned, spontaneously generated precise patterns of activity have been reported in vitro and in vivo [22,23,24], yet for every paper that reports such patterns, there appears to be at least two studies that negate their statistical significance. The fact that these precise patterns can actually be triggered by the experimenter, as shown by Molnár et al. (and also by MacLean et al. with thalamic stimulation [30]), makes the discussion of their statistical significance moot, and reorients the question to the examination of their mechanism and function. Leaving aside the potential function of these precise patterns for another discussion, the data of Molnár et al. could explain how these patterns are generated, by demonstrating that they can be triggered by the firing of single pyramidal neurons. This is a very different scenario from past proposals, which have focused on the synchronous firing of groups of cortical neurons (Abeles’ synfire chains [31]), or on the pacemaker behavior found in subtypes of cortical cells [32]. The demonstration that cortical circuits can generate and propagate precise spatiotemporal patterns of activity, together with the data from Brecht et al. eliciting stereotypical motor patterns by stimulating individual cortical neurons, supports the possibility that the cortex may fundamentally resemble the central pattern generators that dominate motor circuits [33,34], as if the forebrain represented the encephalization of more primitive fixed action patterns [35].

*3. Human differences:* Finally, the data from Molnár et al. reveal strong synaptic pathways in human neocortex. These strong interactions have not been seen before in other species, raising the possibility that human neocortex is endowed with specialized circuit properties. This is a controversial suggestion, since our large prefrontal lobes suggests that mental differences among species are due to differences in the size of cortical circuits, rather than differences in their neurons or modes of operation. Nevertheless, recordings from monkey interneurons have revealed physiological differences from those from rats, as if neurons from the same type were functionally different across species [36,37]. In fact, although chandelier cells have been described in many mammalian species, including marsupials [38], they are particularly complex in humans, with larger axons and morphologically more elaborate cartridges, as compared to those of mice [5,39]. One could argue that these morphological differences may translate into the ability to strongly recruit polysynaptic chains. Or, perhaps morphological differences between chandelier cells are coincidental, and it is the biophysical characteristics of human excitatory synapses onto interneurons that enable these uniquely human circuit properties. Finally, perhaps human cortical circuits have significant physical differences from those from other species, an idea supported by the morphological differences found when comparing the neocortex across different mammalian species (reviewed in [40]).

Further investigations of all these intriguing possibilities could potentially lead us to the essence of our humanity and mental world, or demonstrate, on the other hand, our similarities with other species. In any case, almost a hundred years after it started, the debate between Cajalians and Lorentians as to whether mice are essentially different than men is still open.

## Abbreviations

AAC: axo-axonic cell
EPSP: excitatory postsynaptic potential
IPSP: inhibitory postsynaptic potential
